# Multi-Omics Research Accelerates the Clarification of the Formation Mechanism and the Influence of Leaf Color Variation in Tea (*Camellia sinensis*) Plants

**DOI:** 10.3390/plants13030426

**Published:** 2024-01-31

**Authors:** Yan-Gen Fan, Ting-Ting Zhao, Qin-Zeng Xiang, Xiao-Yang Han, Shu-Sen Yang, Li-Xia Zhang, Li-Jun Ren

**Affiliations:** 1 College of Horticulture Science and Engineering, Shandong Agricultural University, Tai’an 271018, China; fanyangen@sdau.edu.cn (Y.-G.F.); datingsdau@126.com (T.-T.Z.); qzshine@sdau.edu.cn (Q.-Z.X.); hanxy@sdau.edu.cn (X.-Y.H.); 2 Yipinming Tea Planting Farmers Specialized Cooperative, Longnan 746400, China; leegenvan@163.com

**Keywords:** albinism, etiolation, transcriptome, metabolome, anthocyanin

## Abstract

Tea is a popular beverage with characteristic functional and flavor qualities, known to be rich in bioactive metabolites such as tea polyphenols and theanine. Recently, tea varieties with variations in leaf color have been widely used in agriculture production due to their potential advantages in terms of tea quality. Numerous studies have used genome, transcriptome, metabolome, proteome, and lipidome methods to uncover the causes of leaf color variations and investigate their impacts on the accumulation of crucial bioactive metabolites in tea plants. Through a comprehensive review of various omics investigations, we note that decreased expression levels of critical genes in the biosynthesis of chlorophyll and carotenoids, activated chlorophyll degradation, and an impaired photosynthetic chain function are related to the chlorina phenotype in tea plants. For purple-leaf tea, increased expression levels of late biosynthetic genes in the flavonoid synthesis pathway and anthocyanin transport genes are the major and common causes of purple coloration. We have also summarized the influence of leaf color variation on amino acid, polyphenol, and lipid contents and put forward possible causes of these metabolic changes. Finally, this review further proposes the research demands in this field in the future.

## 1. Introduction

The tea plant (*Camellia sinensis*) is a widespread crop with high economic and health potential. Tea plant buds and leaves can be processed into popular non-alcoholic beverages [1]. The primary reasons for tea’s popularity are its heightened metabolites and rich flavors. Among these metabolites, catechins are an expansive class of polyphenols making up 12–24% of the dry weight of young tea leaves, imparting an astringent flavor [2]. Regarding human health, catechins have characteristic antioxidative, anti-inflammatory, and other important biological activities [3]. Theanine (γ-glutamylethylamide), a non-proteinogenic amino acid, is specifically synthesized in tea plants. Generally, theanine is the predominant free amino acid in tea leaves and makes up 1–2% of its dry weight [2]. Theanine has conventional health benefits, including antioxidation and neuroprotection [4]. When drinking tea, theanine is the main flavorful substance related to its sweetness and umami, which can neutralize the astringent flavors of polyphenols and the bitter taste resulting from caffeine [2]. Additionally, tea contains other important functional metabolites, including chlorophylls, carotenoids, lipids, and anthocyanins [5]. The content and ratio of these compounds contribute significantly to the economic and nutritional value of tea plants.

Typically, green-leaf tea varieties are most commonly planted and used to make tea. Recently, increasing numbers of tea mutants with variable leaf colors, including albinism, etiolation, and purple hues, have been used in tea production due to their characterized accumulation of certain bioactive substances and increased economic value [6]. As albinistic or etiolated leaves often lead to a limited photosynthetic capacity and an impaired plant survivability, chlorina tea varieties should adopt special survival strategies. In agricultural practices, certain tea cultivars with periodic chlorina behavior are widely utilized because of their good balance between quality and environmental adaptability [6,7,8,9,10]. For periodic chlorina tea varieties, an albinistic or etiolated phenotype is induced according to specific developmental stages or ecological conditions. For instance, chlorina leaves are observed in *Anjibaicha* (Alternative names: *Baiye No1*, *White leaf No.1*, and *Anji white 1*) and *Huabai 1* under low temperatures [11,12], and high-density light is required for the etiolated phenotype in other varieties such as *Huangjinya* [9,13,14]. When these environmental factors are not satisfied, these varieties will exhibit green leaves to enhance their environmental adaptability [14,15]. In other periodic chlorina tea varieties, leaf re-greening is not modulated by ecological factors but by foliar age [16]. Aside from periodic chlorina cultivars, some varieties with variegated leaves have also been chosen for tea cultivation. In variegated-leaf varieties, a percentage of green tissue is retained on the chlorina leaves, also offering the tea plants an acceptable environmental adaptability [6,17,18]. Alongside changes in leaf color, there are drastic and complex influences on the accumulation of various important functional components in chlorina tea [6,8,9].

Tea varieties possessing purple leaves represent another emerging popular tea type [19]. Compared to traditional green-leaf cultivars, purple-leaf tea is rich in purple-colored bioactive anthocyanins [20,21,22]. Anthocyanins are polyphenols and possess powerful antioxidative and anti-inflammatory capacities [23]. The high-level accumulation of anthocyanins in purple tea hinders the accumulation of other metabolites, predominantly flavonoids [19]. Clearly, enhanced anthocyanin accumulation will greatly alter the flavor of the tea, not just its appearance [19].

As a crop with its leaves acting as the primary economically valuable components, variations in tea leaf color often lead to positive changes in nutritional value and flavor quality. Exploring the metabolites that are associated with leaf color formation and quality changes has theoretical value and important practical value. Therefore, tea is an ideal model plant for investigating the relationship between leaf color and metabolism regulation. Due to the lack of effective gene identification and functional confirmation tools, the cloning of mutated genes for tea leaf color variations is relatively undefined. Fortunately, due to the recent rapid progress of multi-omics technology and its extensive application to tea studies, several high-quality genomes of tea plants have been documented and used to guide physiological and molecular analysis of various important traits [1,24,25,26,27]. To date, over 70 articles have reported the utilization of genome, transcriptome, metabolome, proteome, and lipidome approaches alone or in combination to uncover the color variations in tea. This review will summarize these recent advances and propose challenges for further research.

## 2. Multi-Omics Approaches Further Our Understanding of Leaf Color Variation in Tea

### 2.1. Physiological Mechanisms of Albinism or Etiolation in Tea

Chlorophylls and carotenoids are the two primary pigment types in plant leaves. The biosynthesis and degradation of chlorophylls and carotenoids are complex processes with multiple steps, and chloroplast development is a delicate process. Therefore, from a genetic point of view, the mutations causing chlorina phenotypes in the leaves are very rich [28].

As in all albinistic and etiolated tea mutants, varying degrees of reduced chlorophyll a, chlorophyll b, and total chlorophyll content are the most direct factors causing the leaf color to become lighter (Table 1) [12,16,29,30,31,32,33,34]. The ratio of chlorophyll a/b is down-regulated in *Anjibaicha* [10,35], *Huangjinya* [32,36,37], and *Yanlingyinbiancha* [18] but up-regulated in *Baijiguan* [38], *Xiangfeihuangye* [39], and *Huangjinju* [13]. Additionally, the content of carotenoids in chlorina leaves is also always lower as compared to green-leaf controls [10,13,14,16,18,29,30,32,33,34,39,40,41,42]. There are exceptions, however: for example, the chlorina tea shoot (HY) of *Danzicha* accumulates 1.84 times higher levels of carotenoids than the green shoot from the same tree [43]. The ratios of chlorophylls to carotenoids are decreased in all chlorina tea plants except in the HY shoot of *Danzicha* [10,13,14,16,18,29,30,32,33,34,39,40,41,42]. These findings suggest that the absolute and relative decreases in total chlorophyll content across most chlorina tea plants are much higher than for carotenoids, and the resulting leaf color appears yellowish. As chlorophyll and carotenoid accumulation are highly interdependent, it is challenging to determine whether the reduction in chlorophyll or carotenoid content is the fundamental cause of most chlorina tea plants’ formation [44,45].

Targeted metabolomic methods are often used to analyze the changes in carotenoid composition and contents in chlorina leaves. In tea plants, lutein is the most prevailing carotenoid component, followed by neoxanthin, violaxanthin, cryptoxanthin, α-carotene, β-carotene, and zeaxanthin [10]. Compared to green-leaf varieties, the levels of lutein, neoxanthin, and violaxanthin in the chlorina leaves of *Anjibaicha* and *Huangjinya* are dramatically reduced, while the levels of α-carotene, β-carotene, and zeaxanthin are increased [10,32,37]. A different effect of the chlorina phenotype on carotenoid accumulation is observed in *Huangjinshuixian*, *Yujinxiang*, and *Huangyu*. In these three varieties, the contents of lutein, carotene, neoxanthin, and cryptoxanthin are all down-regulated [16,30,33]. The levels of β-carotene in *Zhonghuang 2* are also reduced [42]. An enhanced accumulation of zeaxanthin is consistently observed in *Zhonghuang 2*, *Yujinxiang*, and *Huangyu*, but not in *Huangjinshuixian* [16,30,33,42]. These findings indicate that the metabolic flow allocation in the carotenoid pathway varies greatly in different chlorina tea plants. This is logical as carotenoids are important photo-protective pigments in plants. Blocking photosynthetic electron transfer in chlorina leaves is more prone to producing excess reactive oxygen species and radicals under high light or UV stress. The efficacy of different carotenoids in scavenging various reactive oxygen species varies greatly [54]. For example, β-carotene has a high activity in ^1^O_2_ quenching, while lutein and α-carotene exhibit a much lower efficacy [55]. For radical scavenging and UV protection, zeaxanthin is more efficient than β-carotene [56,57]. Albino leaves may enhance the metabolic flow toward more highly active carotenoid components to enhance antioxidative abilities when the total carotenoid content decreases.

### 2.2. Molecular Mechanisms of Leaf Chlorosis in Tea Plant

Cloning the mutated gene responsible for albinism or etiolation in tea varieties is the most important, but still challenging, approach to studying the formation of leaf chlorosis. To date, only two studies have identified highly reliable candidate genes for leaf chlorosis in tea plants via omics techniques [24,38]. Using genotyping-by-sequencing, Zhang et al. constructed a genetic map of the full-sibling population of *Baijiguan* and *Longjing43* and successfully identified the major effect QTL linked to the total chlorophyll contents [38]. Using bulked segregant analysis sequencing (BSA-Seq)-assisted genetic mapping, a nonsynonymous mutation in the magnesium chelatase I subunit encoding the gene *CsChlI* blocks the conversion of protoporphyrin IX (Proto IX) into MgP IX throughout chlorophyll synthesis, impacting leaf coloring [38]. In many annual plants, similar non-synonymous mutations are reported to be responsible for the reduced function of ChlI and chlorophyll accumulation [58,59,60,61]. A common feature of these mutants and *Baijiguan* is that the degree of leaf yellowing is gene-dosage-dependent [58,59,60].

In another study, a pan-genome analysis of 22 tea accessions revealed various genomic deletions in the *glutamyl-tRNA synthetase* (*GluRS*/*EARS*) gene of both *Anjibaicha* and *Huangjinya* [24]. GluRS/EARS catalyzes the formation of L-Glu-tRNA, which is considered the first step of chlorophyll biosynthesis in higher plants [62]. The truncated GluRS/EARS protein may limit chlorophyll synthesis and contribute to the formation of the chlorina phenotype in these two varieties. Additionally, structural variations (SVs) have been identified in another three chlorophyll synthesis genes (*chlorophyllide a oxygenase*, *CAO*; *geranylgeraniol reductase*, *CHLP*; *glutamyl-tRNA reductase*, *GluTR*) and one (*magnesium chelatase D subunit*, *ChlD*) chlorophyll synthesis gene in *Huangjinya* and *Anjibaicha*, respectively. These findings suggest the presence of multiple blocked genetic sites in the chlorophyll synthesis pathways of *Anjibaicha* and *Huangjinya* [24]. Additionally, the genomic region of the chlorophyll-degradation-related gene *chlorophyll b reductase* (*NOL*) has a 1-base-pair deletion in *Anjibaicha*, suggesting that the chlorophyll degradation may also have changed [24,63,64]. Comparative genomic analysis identified a genomic variation in the *CYP97A3* gene causing a 20-amino-acid alteration that may affect its ability to catalyze zeaxanthin synthesis of lutein [24,65]. However, the true functions of the aforementioned candidate genes identified using either genetic mapping or pan-genome analysis remain unclear in vivo.

**Figure 1:**
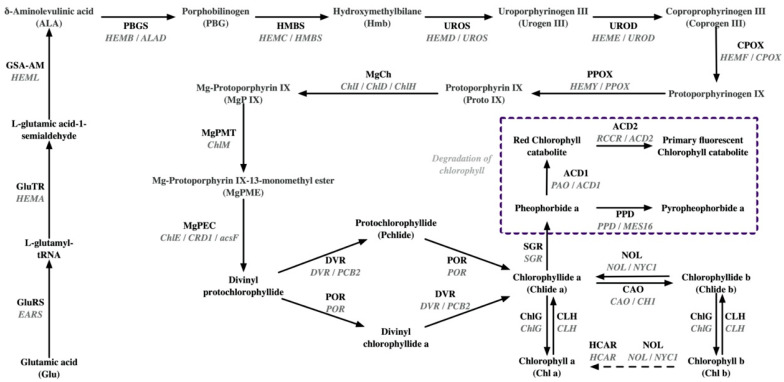
Proteins and genes involved in chlorophyll synthesis pathway. Next to the arrow, the word above represents the protein name, and the corresponding gene name is below. GluRS, glutamyl-tRNA synthetase; GluTR, glutamyl-tRNA reductase; GSA-AM, glutamate-1-semialdehyde 2,1-aminomutase; PBGS, porphobilinogen synthase; HMBS, hydroxymethylbilane synthase; UROS, uroporphyrinogen-III synthase; UROD, uroporphyrinogen decarboxylase; CPOX, coproporphyrinogen III oxidase; PPOX, protoporphyrinogen III oxidase; MgCh, magnesium chelatase; MgPMT, magnesium-protoporphyrin O-methyltransferase; MgPEC, magnesium-protoporphyrin IX monomethyl ester (oxidative) cyclase; DVR, divinyl chlorophyllide a 8-vinyl-reductase; POR, protochlorophyllide reductase; NOL, chlorophyll(ide) b reductase; CAO, chlorophyllide a oxygenase; ChlG, chlorophyll/bacteriochlorophyll a synthase; CLH, chlorophyllase; HCAR, hydroxymethyl chlorophyll a reductase; SGR, magnesium dechelatase; PAO/ACD1, pheophorbide a oxygenase; ACD2, red chlorophyll catabolite reductase; PPD, pheophorbidase.

Given the difficulty of identifying mutated genes at the genomic level, much of the research in this field uses metabolomes, proteomes, and transcriptomes to indirectly investigate the molecular mechanisms underlying leaf chlorosis in tea. A joint assessment of proteomes and metabolomes revealed one potential blocked site of chlorophyll synthesis in etiolated leaves of *Huangjinya*: from protochlorophyllide (Pchlide) into chlorophyllide a (Chlide a). The significantly lowered protein abundance of protochlorophyllide oxidoreductase (POR) in the etiolated leaves was considered to be responsible for the excessive accumulation of the substrate (MgP IX) in the previous step [9]. While the metabolic flow from coproporphyrinogen III (Coprogen III) to Proto IX aligns with the trend of the excessive accumulation of substrates and a reduced product content, the abundance of protein (coproporphyrinogen III oxidase, COPX/HEMF) mediating this catalytic reaction does not change in etiolated leaves [9]. The alterations in the content of Coprogen III and Proto IX are more likely influenced by other metabolic events. In addition, a dramatically lower level of CAO may further decrease the content of chlorophyll b (Figure 1) [9]. Pheophorbide a oxygenase (PAO/ACD1)-mediated chlorophyll breakdown is essential for the loss of green pigments in plant leaves [66]. Overactivated PAO/ACD1 in *Huangjinya* may enhance the chlorina phenotype. In the etiolated leaves of *Huangjinya*, proteome analysis demonstrates that the function of the photosynthetic chain is greatly damaged [9,36]. Fan et al. reported that most protein members of Photosystem I (PSI), PSII, and plastid quinone pool (PQ) are significantly down-regulated in yellowish leaves [9]. In another study, differentially expressed protein (DEP) analysis also indicates an impaired photosynthetic chain, but the DEPs identified are less repetitive and reduced in number [36].

Proteome, acetyl-proteome, and succinyl-proteome analysis in *Anjibaicha* uncovered an enrichment of differentially expressed or modified proteins in the photosynthetic pathway during the whitening and re-greening processes of *Anjibaicha*, suggesting that the destruction and reconstruction of the photosynthetic chain function is closely associated with the change in leaf color [8,46,67]. In support of this, transcriptomic analysis also identified enriched down-regulated differentially expressed genes (DEGs) in the photosynthetic chain and chlorophyll metabolism, respectively, including eight and six genes [35]. Among them, the antenna protein LHCA4 exhibits a consistently lower abundance in etiolated leaves at the transcriptional, protein, and protein modification levels [8,35,46].

In most other albinistic or etiolated varieties, the evidence at the protein or transcriptome level supports one or more of the processes of chlorophyll metabolism, the photosynthetic chain, and carotenoid synthesis being impacted in chlorosis leaves (Table 1). Understandably, blocked biosynthesis of chlorophyll and carotenoids and activated chlorophyll degradation contribute to the lightened color of the leaves [68,69]. While not all genes in the biosynthetic pathways of chlorophyll and carotenoids are uniformly down-regulated (Table 1), the reduced expression of crucial upstream genes may explain the decreased accumulation of total chlorophyll or carotenoids in *Anjibaicha* (*POR* and *PBGD*/*HMBS*/*HEMC*) [35] and the albinistic branches of *Huangshan* (*CHLP* and *POR*) [48], *Huangjinshuixian* (*DXS* and *GGPPS*) [16], and *Zhonghuang 3* (*GluTR*/*HEMA*, *GSA-AM*/*HEML*, *UROD*/*HEME*, *HEMF*/*CPOX*, and *CHLP*) [14].

In plants, the biosynthesis and degradation of chlorophyll are highly coordinated with the structural and functional integrity of the photosynthetic chain [28]. The LHC proteins associated with the antenna complex are responsible for light harvesting and Chl a/b binding [70]. Incompletely assembled photosystems will hinder the localization of antenna proteins to the thylakoids and will cause reduced contents of LHC-bound chlorophyll [71]. As free chlorophyll is photosensitive, the absence of LHCB1 and LHCB2 can lead to reduced chlorophyll accumulation [72,73]. In various chlorina tea varieties, the functional modules (antenna complex [8,9,13,29,30,40,41,48,52,53], photosystems [8,9,12,17,48,53], quinone pools [36], cytochrome b6/f complexes [9,40], and ATPase complexes [8,9,48,53]) of the photosynthetic chain have been differentially impaired (Table 1). The reduction in antenna protein abundance and the photooxidative stress triggered by photosynthetic electron transfer defects may be common factors leading to or exacerbating leaf chlorosis in these tea variations [74].

Reduced chlorophyll content will disrupt photosynthetic chain stability. Light-induced thylakoid biogenesis is a prerequisite for the assembly of the photosynthetic chain, which is highly coordinated with the reduction of Pchlide into chlorophyllide (Chlide) in the penultimate step of chlorophyll biosynthesis [73]. In addition, the protein stability of the LHCII members is modulated by chlorophyll [64]. In *Arabidopsis cao1* mutant chloroplasts, down-regulated chlorophyll b promotes the proteolysis metabolism of the LHCII proteins [75]. Therefore, it is still difficult to distinguish whether a reduced chlorophyll content or an impaired photosynthetic chain is more likely to cause leaf chlorosis in tea according to the available omics results.

Multi-omics technologies have identified potential transcriptional factors involved in regulating the chlorophyll metabolism in chlorina tea plants [16,39,50,51]. Both transcriptome and translatome analyses reveal that *ELONGATED HYPOCOTYL 5* (*HY5*) (Figure 1), a bZIP-type transcription factor (TF) involved in photomorphogenesis, is up-regulated in *Xiangfeihuangye* [39]. HY5 serves a conserved role in negatively regulating chlorophyll synthesis [76]. Gene expression analysis has uncovered that *HY5* is negatively correlated with genes associated with light harvesting and chlorophyll biosynthesis in tea plants. The expression of HY5 is inhibited, thereby relieving its inhibition of chlorophyll synthesis during the seasonal re-greening of *Xiangfeihuangye* [39]. During the seasonal re-greening of *Huangkui*, the positive regulation of genes related to light harvesting and chlorophyll biosynthesis due to increased *REVEILLE1* (*RVE1*) expression has been confirmed by transcriptome findings and other molecular biological results [51]. In the future, it will be necessary to determine whether there are genomic variations in these TF genes and characterize their genetic contributions to the formation of chlorina phenotypes in tea plants.

### 2.3. Physiological Mechanisms of Purple Leaf Coloration in Tea

Two main types of purple coloring behavior have been documented in tea plants: at the developmental stage and due to ecological-factor-induced purple pigment accumulation [19]. In purple varieties such as *Zijuan* [77], *Zixin* [78], and *Wuyiqizhong18* [79], purple pigments accumulate in the young leaves and stems but not in mature tissues. In some ecologically dependent varieties, such as *Longjing43* [80] and *TRFK 306* [81], the biosynthesis of purple pigments is triggered by environmental stress, such as temperature, water, and light.

**Figure 2:**
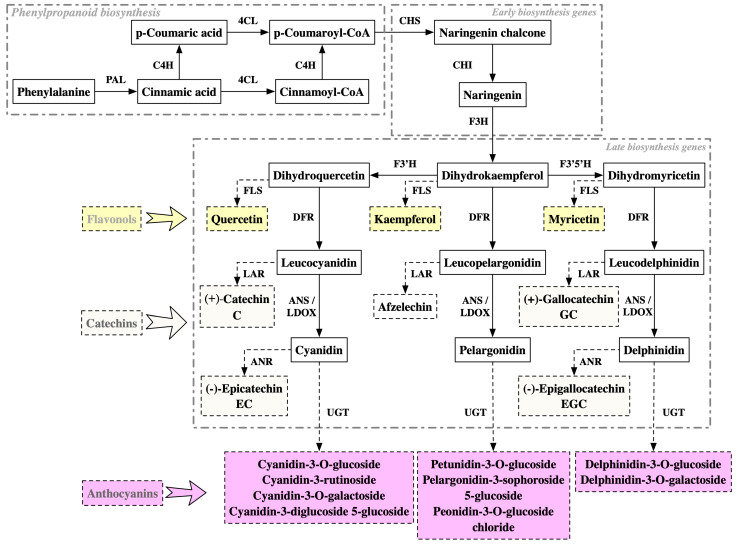
The synthesis process of flavonoids and anthocyanins. The yellow textboxes represent the flavonols, the pale green textboxes represent the catechins, and the purple textboxes represent the decorated anthocyanins. PAL, phenylalanine ammonia lyase; C4H, cinnamate-4-hydroxylase; 4CL, 4-coumarate: CoA ligase; CHS chalcone synthase; CHI chalcone isomerase; F3H, flavanone 3-hydroxylase; F3′H, flavonol 3′-hydroxylase; F3′5′H, flavonol 3′5′-hydroxylase; FLS, flavonol synthase; DFR, dihydroflavonol 4-reductase; LAR, leucoanthocyanidin reductase; ANS/LDOX, leucoanthocyanidin dioxygenase; ANR, anthocyanin reductase; UGT, UDP-glucose: flavonol-3-O-glycosyltransferase.

Numerous studies have determined that water-soluble anthocyanins are the primary purple pigments in tea [19]. As illustrated in Table 2, glycosylated or acylated derivatives of cyanidin, delphinidin, petunidin, pelargonidin, peonidin, and malvidin can be detected in purple tea leaves [77,78,80,82,83,84,85]. Among these metabolites, only cyanidin-3-O-galactoside is the major anthocyanin composition in nearly all the studied purple tea varieties [77,78,80,82,83,84,85]. Apart from cyanidin-3-O-galactoside, delphinidin-3-O-galactoside, cyanidin-3-O-glucoside, cyanidin-3-rutinoside, and delphinidin-3-O-glucoside are another four anthocyanin components in tea identified with the second highest frequency in various environments and different varieties (Figure 2) [81,83,84].

Besides the genetic factors, environmental variations also influence the anthocyanin composition [93]. For instance, in the most studied purple variety, *Zijuan*, delphinidin-3-O-galactoside is detected at high levels in five different geographical environments but not in ‘Pu’er’ [77,82,87,88,89,91]. The components of anthocyanin identified also depend on the detection strategy. In the study conducted by Tan et al., a total of 45 differentially accumulated anthocyanins were detected using targeted UPLC–ESI–MS/MS analysis in *Zijuan*, *Ziyan*, and *Chuanzi* [82]. This study identified the most diverse types of anthocyanins in *Zijuan* [82]. In addition, both of the two most abundant anthocyanins, cyanidin-3-O-galactoside and delphinidin-3-O-galactoside, cannot be detected in the steamed tea of *Zijuan*, suggesting that tea processing also influences the output of anthocyanin composition analysis substantially [82,86].

### 2.4. Molecular Mechanisms of Purple Coloration in Tea Leaves

Multi-omics studies have uncovered a range of differentially expressed genes or differential alternative splicing genes linked to anthocyanin biosynthesis, anthocyanin transportation, anthocyanin degradation, and transcriptional regulation (Table 3). In flowering plants, MYB TFs assemble into a well-known ternary complex MBW with members of the WD40 and bHLH families to modulate the expression of various structural genes involved in anthocyanin biosynthesis [94,95]. The activation of R2R3-MYB transcription factors, including *AN1*/*MYB75* [82,96], *MYB114* [24], and *MYB90* [84], is documented in most studies related to purple tea, suggesting that the MBW complex may possess a common and conserved transcriptional regulatory role in tea anthocyanin synthesis. Consistently, the molecular experimental evidence has verified this hypothesis. Performing the overexpression of *MYB90* from *Zikui* in the calluses of tobacco can strongly induce anthocyanin synthesis [84]. Moreover, Sun et al. reported that AN1/MYB75 could physically interact with bHLH TFs (GL3 and EGL3) to recruit the WD40 protein TTG1 to induce a large amount of anthocyanin accumulation in *Nicotiana benthamiana* leaves [77]. At the genomic level, a recent study has identified a 148 bp LTR/*Gspsy* insertion into the upstream region of *MYB114* in *Zijuan*, *Jinguanyin*, and *Jinmingzao*, which provides potential genomic clues supporting the high expression of the *MYB* gene in these purple varieties [24].

Anthocyanin biosynthesis begins with phenylalanine and is catalyzed by a series of enzymes involved in phenylpropanoid metabolism, the early flavonoid biosynthesis process, and the late flavonoid biosynthesis process [77]. The transcriptome and proteome data reveal a consistent up-regulation of the late biosynthesis gene (LBG) *anthocyanin synthase* (*ANS*) in all examined purple tea leaves (Table 3). ANS/LDOX catalyzes colorless leucoanthocyanins to form anthocyanidin, operating as the penultimate step of anthocyanin biosynthesis [100]. Potential binding sites of MYB transcription factors (TFs) are identified in the promoter regions of *ANS*/*LDOX* genes. Yeast one-hybrid assays have verified that the MYB TF AN1 can directly bind to the promoter of ANS/LDOX genes, indicating that ANS/LDOX plays a vital role in the MBW-regulated anthocyanin synthesis pathway [77].

Additionally, the up-regulation of certain other genes classified as LBGs, including flavonoid-3′-hydroxylase (F3′H), flavonoid 3′,5′-hydroxylase (F3′5′H), dihydroflavonol-4-reductase (DFR), and UDPG-flavonoid glucosyltransferase (UGT) are identified in the purple leaves of some tea varieties. However, the specific gene members identified vary from study to study (Table 3) [77,78,80,82,83,84,85]. A similar gene expression change behavior is observed for the genes involved in the substrate competition pathways in anthocyanin biosynthesis (Table 3). In particular, flavonoid synthase (FLS), leucoanthocyanidin reductase (LAR), and anthocyanidin reductase (ANR) are responsible for catalyzing delphinidin, leucodelphinidin, and delphinidin to produce flavonols, catechin, and epicatechin, respectively [77]. The deactivation of one or more metabolic substrate competition pathway in anthocyanin biosynthesis is differentially observed in various purple-leaf varieties [80,82,89,90,99].

Additionally, the multi-omics data have identified several specific auxiliary mechanisms of anthocyanin accumulation in the purple leaves of various tea varieties. For example, in *Zijuan* and *Zikui*, the down-regulated expression of genes associated with anthocyanin degradation (*Polyphenol oxidase*, *PPO*) could contribute to anthocyanin accumulation (Table 3) [84,89]. The activated transportation of anthocyanins into the vacuoles can limit their degradation and help to increase their accumulation [101,102]. *Zijuan* and *Chuanzi* each have an up-regulated anthocyanin transporter protein, ABC transporter B8 [20] and glutathione S-transferase, respectively [82]. The gene expression changes in these anthocyanin transporters in response to ecological factors are consistent with the change trends in the anthocyanin content [81,96]. Therefore, protecting anthocyanin from degradation may be an essential synergistic mechanism to increase the accumulation of anthocyanins in purple tea leaves.

## 3. Metabolic Reprogramming by Leaf Color Variations in Tea Plants

### 3.1. Influence of Chlorina Variations on Amino Acid Metabolism

**Figure 3:**
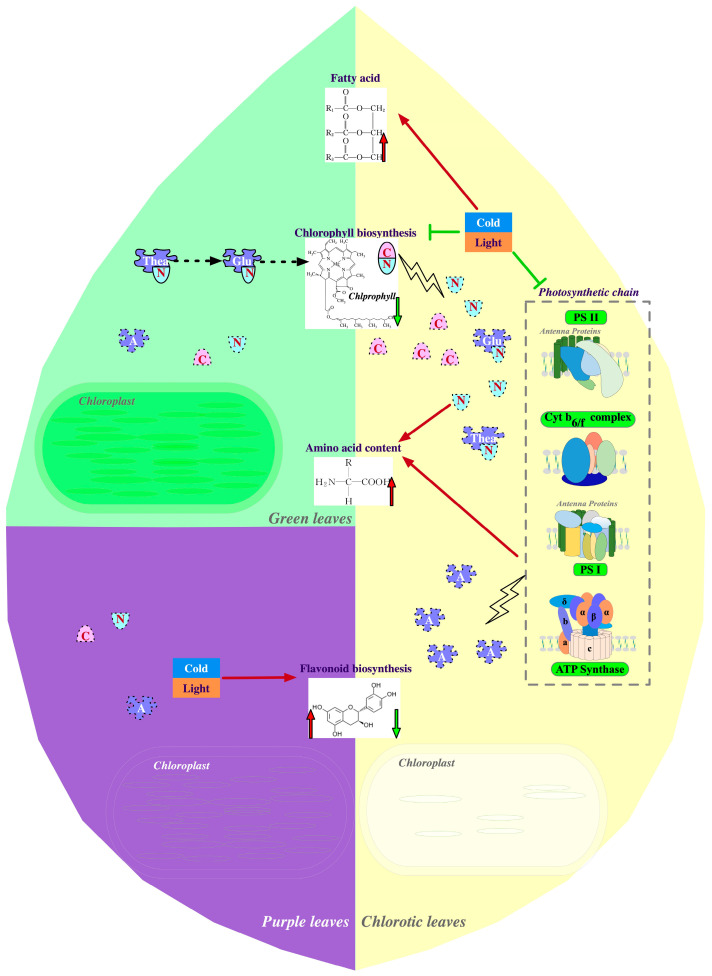
Red/green frameless arrows represent a promoting/inhibitory effect, while red/green framed arrows represent an increase/decrease in substances. Substances surrounded with a dashed outline are in a free state. Compared to green varieties, chlorotic varieties are hindered in chlorophyll synthesis and degradation under strong light or low-temperature conditions, resulting in imbalanced carbon (C) and nitrogen (N) metabolism and reduced flavonoid content in the chlorotic leaves. Excess free nitrogen promotes the accumulation of free amino acids in chlorotic leaves. On the other hand, the loss of chloroplast structure and degradation of photosynthetic-chain-related proteins further increase the content of free amino acids in chlorotic leaves. In addition, the synthesis of fatty acids has also increased in chlorotic varieties. In purple varieties, low temperature or strong light enhances the expression of flavonoid-pathway-related genes, promoting the synthesis of more anthocyanins.

In the albinistic or etiolated leaves of chlorina tea varieties, the formation of certain flavors is closely related to the changes in tea theanine and polyphenol levels. Generally, the content of total free amino acids is much higher in chlorina leaves than in green leaves from re-greening or green-leaf plants (Figure 3) [32,40,43,48,49,52,53,103]. As the most abundant free amino acid in tea leaves, alteration in the absolute content of theanine contributes heavily to the change in the total free amino acid [32,40,43,48,49,52,53,103]. Additionally, glutamic acid, glutamine, and aspartic acid are three other free amino acids with high levels after theanine in tea leaves. The accumulation of these three amino acids is also dramatically enhanced in chlorina leaves (Figure 3) [32,40,43,48,49,52,53,103].

However, not all chlorina variations demonstrate an elevated accumulation of free amino acids. For instance, the levels of total free amino acids in the etiolated leaves of *Huangjinju* only increase slightly compared to re-greening leaves, and the differences do not reach the significance level [13]. In another chlorina variety, *Baijiguan*, the levels of free amino acids in the etiolated leaves are not always higher than that in the green-leaf control, *Longjin43*, and the conclusions drawn can even be the opposite [104]. In addition, no significant differences are observed in the total amino acids between the etiolated and green half-siblings generated from *Baijiguan* and *Longjin43* [15]. One possible explanation for these findings, which contradict our understanding that chlorina enhances the accumulation of free amino acids, especially theanine, is that the change in amino acid accumulation in some green-leaf varieties is more drastic than that in chlorina varieties under specific environments. One piece of evidence that supports this hypothesis is that the levels of theanine in the etiolated variety *Yujinxiang* are higher and lower than that in *Shuchazao* under non-shaded and shaded conditions, respectively [33].

The aforementioned clues indicate that the changes in theanine content and total free amino acids are consistent in most cases [32,40,43,48,49,52,53,103]. Therefore, many studies mainly focus on the molecular mechanisms underlying the differences in theanine content among different leaf color materials. The current multi-omics evidence mainly provides three plausible explanations for why leaf chlorosis can elevate the theanine levels in albinistic or etiolated tea varieties. The first possible explanation may be that the expression levels of the genes involved in theanine synthesis or degradation are activated or repressed (Figure 3). Glutamate and ethylamine serve as precursors of theanine biosynthesis in tea plants, and this enzyme reaction is catalyzed by theanine synthetase (TS), a member of the glutamine synthetases (GSs) [2]. The other GS members may also have a certain TS activity [105]. In *Anjibiacha*, *HY*, and *Zhonghuang 3*, transcriptome analysis has demonstrated that the expression levels of some TS or GS genes are positively correlated with the theanine content, suggesting enhanced TS activity in these varieties [14,43,48,52,106]. In addition, the up-regulation of glutamate synthase (GOGAT), glutamate dehydrogenase (GDH), alanine decarboxylase (AlaDC), and alanine transaminase (ALT) in chlorina leaves may increase the abundance of glutamate and ethylamine and promote theanine biosynthesis [42,49,52,106]. In *Zhonghuang 3*, the mRNA abundance of two genes involved in theanine hydrolysis, *PDX2* and its homolog *glutamine amidotransferase 5* (*GGP5*), is significantly down-regulated in the etiolated leaves, potentially influencing theanine accumulation [49,107].

Theanine is not only the most abundant free amino acid in the tea plant but also acts as the primary nitrogen storage source. A second possible reason for an increased theanine content is reduced nitrogen consumption in chlorina leaves. In higher plants, the theanine synthesis substrate glutamate plays a central hub role in nitrogen metabolism and is an important link connecting the carbon and nitrogen metabolic balance [108]. Importantly, the photosynthetic pigment chlorophyll’s biosynthesis depends on glutamate as the nitrogen source. A much lower chlorophyll content in chlorina tea leaves may decrease the nitrogen consumption, contributing to a higher glutamate accumulation. Another indirect piece of evidence supporting this hypothesis is the observation of an increased NH_4_^+^ content in chlorina leaves [16,32]. As the biosynthesis of glutamate is a primary pathway that consumes NH_4_^+^, the accumulation of ammonium ions also supports repressed nitrogen utilization in albino leaves [32]. Based on this, we speculate that the reduction in the use of glutamic acid, an important organic nitrogen source, causes more nitrogen to be stored in the form of theanine in chlorina tea leaves.

Thirdly, the activation of protein degradation may be a potential cause of highly accumulated free amino acids, including theanine, in chlorina tea leaves [16,18,32,50,52]. Via transcriptome analysis, a group of up-regulated genes involved in the ubiquitin–proteasome system has been identified in chlorina tea leaves [16,18,32,50,52]. Specifically, the expression levels of associated genes encoding E3 ubiquitin ligase, ubiquitin, damaged DNA binding protein, and ubiquitin-specific protease are enhanced to varying degrees in *Yanlingyinbiancha* and *Fuhuang 1* [18,52]. In *Koganemidori*, the autophagy pathway, critical to organelle degradation, is also up-regulated, indicating that abnormally developed chloroplasts may be directed to the recycling system [50]. The aforementioned significant decrease in protein abundance of the photosynthetic chain may be attributed to the combined effects of protein degradation and chloroplast recycling. Accordingly, more free amino acids will be released.

### 3.2. Effects of Chlorina Variation on Flavonoid Metabolism

Tea polyphenols are an expansive group of flavonoids, including flavanols, flavanones, flavonols, leucoanthocyanidins, and anthocyanins [109]. In non-purple tea, flavanols represent the most abundant flavonoids (accounting for 60–80%) and are mainly composed of catechins.

Metabolome analysis reveals that the total flavonoids in chlorina varieties, including *Anjibaicha*, *Huangjinya*, *HY*, *Xiangfeihuangye*, *Huangjinju*, and *Zhonghuang 3*, are down-regulated [10,13,37,39,43,49]. At the molecular level, down-regulated expression levels of genes involved in phenylpropanoid biosynthesis and early biosynthesis pathways (Figure 2) may limit the metabolic flow to flavonoid biosynthesis. For instance, the transcript abundances of *PAL* in *Zhonghuang 3*, *CHI* in *Zhonghuang 3* and *Yujinxiang*, *CHS* in *HY* and *Yujingxiang*, and *F3H* in *Xiangfeihuangye* and *Yujingxiang* 1 are positively related to flavonoid contents. In *Huangjinya*, decreased levels of PAL and 4CL proteins have been detected using proteome analysis [36]. Genes in the phenylpropanoid metabolic pathway are not only regulated at the transcriptional level but also in terms of protein modification in *Anjibaicha* [8,10].

Because flavonoids account for over 10% of the dry matter in tea, flavonoid biosynthesis consumes a large number of photosynthetic products. Impaired photosynthesis in chlorina leaves may result in an insufficient carbon skeleton supply for flavonoid synthesis [37,39]. One compelling piece of evidence that supports this hypothesis is that decreased tea catechins are observed in the albinistic branches of normal tea plants but the expression levels of genes in the associated synthesis pathway are unaffected [48]. The lack of a carbon source may be a common cause for the limited polyphenol synthesis capacity in chlorina leaves.

There are some exceptions where the flavonoid content in chlorina varieties unexpectedly increases [19]. In *Yanlingyinbiancha*, a significantly higher catechin content is detected in albinistic tissues but not green tissues. The expression levels of genes involved in phenylpropanoid biosynthesis (*4CL*) and the early biosynthesis pathways (*CHI*) are up-regulated, consistent with elevated flavonoid contents. On the other hand, the up-regulation of *sucrose synthase* in the albinistic tissues of variegated leaves suggests that it may be able to supplement carbon sources from the surrounding green tissues [18]. Therefore, this counterexample supports the high-level expression of upstream structural genes and sufficient carbon sources, which are determinants for ensuring the normal synthesis of flavonoids in tea.

Comparative omics examinations show that differentially accumulated flavonoid compositions between chlorina and green leaves are complex and vary with the tested tea variety and growing environment [15,33,37,39,43]. For instance, the relative flavonol content in the albinistic leaves of *Anjibaicha* exhibits opposite trends across two independent studies, suggesting that the accumulation of certain flavonoids may be more impacted by environmental factors [10,103]. In most chlorina tea varieties, epigallocatechin (EGC) is a flavonoid component with roughly consistent content changes relative to the total flavonoid contents [10,13,15,43,50]. Causally, EGC and its gallate derivative (EGCG) account for most catechins and are more sensitive to changes in the supply levels of carbon sources.

At the molecular level, the expression changes in LBGs, such as *ANS*, *ANR*, and *FLS*, can only explain the changes in metabolic flux toward different subclasses of flavonoids (mainly catechins and flavonols) but cannot explain the changes in the contents of a single component within them [10,13,18,37,39,43,49]. One hypothesis is that the resistance of chlorina leaves to light stress is reduced, which may activate a stronger oxidative stress defense response and promote the accumulation of flavonoids with high antioxidant activity. In plants, some flavonoids containing dihydroxylated B-rings (e.g., quercetin) are accumulated in response to intense sunlight [110]. Also, in the chlorina leaves of both *Huangjinya* and *Yujinxiang*, the contrarian accumulation of quercetin supports the idea of an adaptive synthesis of flavonoids [33,37].

### 3.3. Effects of Chlorina Variations on Fatty Acid Metabolism

Leaf chlorosis also greatly influences the accumulation of lipids in tea [39]. In plants, lipids are critical backup energy storage substances [111]. Given that the accumulation of photosynthetic products is considerably damaged in chlorina leaves, these mutated tea variations may invoke fatty acids to elevate the supply of carbon sources by enhancing the activities of critical enzymes in lipid reuse. Transcriptional analysis has demonstrated that several genes involved in the β-oxidation of fatty acids are up-regulated in the albinistic tissues of *Yanlingyinbiancha*. Among these up-regulated DEGs related to fatty acid oxidation, *acyl-CoA oxidase* (*ACX*) and *peroxisomal 3-ketoacyl-CoA thiolase* (*PKT*) are responsible for the first and final steps of the β-oxidation process in plants [18,112,113], supporting the idea of enhanced energy release with fatty acids in albinistic tissues [18].

Another critical biological function of lipids is as the main components of chloroplast membranes [114]. Monogalactosyldiacylglycerol (MGDG) and digalactosyldiacylglycerol (DGDG) represent the two primary fatty acids in the lipid bilayer of the thylakoid membrane. An adequate ratio of MGDG to DGDG is necessary to maintain a stable thylakoid membrane [115]. In both *Arabidopsis* and soybeans, the MGDG:DGDG ratios are both approximately 2 [115,116]. And the composition of lipids in the chloroplast membrane is highly dynamic and can be modulated rapidly in response to environmental changes [116,117]. While in the etiolated leaves of *Baijiguan*, the ratio of MGDG to DGDG is relatively low, potentially supporting an observed damaged thylakoid membrane [111,118]. When subjected to shade treatment, the MGDG:DGDG ratio exhibits a trend of slightly increasing first and then decreasing. Although the differences among these shaded groups are not significant, the rapid response of the MGDG:DGDG ratio to shading may partially explain the improvement in chloroplast stability during the early re-greening stage [118]. In addition, more polar lipids are accumulated in the etiolated leaves of *Baijiguan* compared to re-greening leaves [118]. Similarly, the polar lipids involved in cell membrane assembly are significantly up-regulated in another etiolated mutant, *Huangjinshuixian*, compared to its green-leaf parent [16]. The findings of these two comparative analyses contradict the idea of chloroplasts acting as lipid synthesis locations, as the chloroplast function is more robust in the green leaves. According to limited information, we speculate that increased polar lipids may be a survival strategy for chlorina plants under normal light conditions. While reducing the light intensity down-regulates the expression of genes associated with polar lipid accumulation [118], the recovery of chloroplast function is insufficient to enhance polar lipid synthesis.

Epidermal wax is another crucial lipid type used by plant leaves to adapt to environmental stress [119,120]. In response to UV-B stress, *HY5* is activated and promotes the biosynthesis of epidermal wax by repressing the expression of a related negative regulator, *DEWAX*, in *Arabidopsis*. *HY5*-mutated or *DEWAX*-overexpressing plants are more sensitive to UV-B irradiation stress, supporting the idea of physical wax barrier protection in UV resistance [121]. Compared to green-leaf tea varieties, significantly increased epidermal wax is detected in *Huangjinshuixian* [15]. At the transcriptional level, increased expression of the *long-chain acyl-CoA synthetase* gene supports the activation of epidermal wax biosynthesis in the albinistic tissues of *Yanlingyinbiancha* [18]. These findings suggest that chlorina tea plants may adjust their lipid metabolism to enhance their adaptability to environmental stress.

### 3.4. Metabolic Changes in Flavonoids Other Than Anthocyanins in Purple Tea

As the primary accessory pigments, anthocyanins are the most well-documented flavonoids in purple tea. Along with enhanced anthocyanin biosynthesis, the levels of other flavonoid compositions have undergone substantial alterations in purple tea [21,78,86,89,91]. Unlike the significant increase in anthocyanins, the changing trend in the content of other flavonoids in purple tea relies on the control chosen for comparative analysis. For example, it is challenging to obtain similar findings when comparing the same purple variety with different green-leaf varieties. For catechins, the purple leaves of *Zijuan* exhibit significantly lower levels of C, GC, and EGCG and similar levels of EC, EGC, and ECG when compared to the green-leaf variety *Yunkang* [89]. Compared to another green-leaf tea variety, *Fudingdabai*, no significant difference in the contents of EC and significantly decreased EGCG contents are also observed in *Zijuan* [82]. However, the trends in the relative content change in EGC and ECG are quite different between these two different comparison group selection strategies [82,89]. The differences in differential metabolites are easy to understand because the accumulation behaviors of flavonoids across different green-leaf controls may also be different. Nevertheless, the consistent result between these two studies is that the total catechin in *Zijuan* is reduced compared to in *Fudingdabai* and *Yunkang* [82,89]. At the molecular level, the significantly lower level of *LAR* (Figure 2) in *Zijuan* may explain the decrease in metabolic flow toward catechin biosynthesis (Table 3) [89].

The levels of these non-purple flavonoids are also compared for two other groups of tea varieties, namely *Jinmingzao* compared to *Huangdan* and *Zikui* compared to *N61* [83,84]. The total catechin levels are higher in *Jinmingzao* and lower in *Zikui* when compared to the corresponding green-leaf variety [83,84]. Furthermore, the proanthocyanidin content is much lower in *Zikui* [84]. However, the opposite result is reported in another study, where monomeric catechin is lowered in purple tea varieties but the proanthocyanidin content increases [91]. In another comprehensive comparative study of nine purple tea varieties and three green-leaf tea varieties, a much higher level of catechins is more likely to be detected in purple tea [85]. These inconsistent results suggest the importance of selecting appropriate green-leaf controls when exploring metabolic alterations in purple leaves.

There are several studies comparing the metabolic differences between tender purple leaves and purple faded mature leaves [78,99,122]. In the purple tea variety *Wuyiqizhong18*, the contents of both total polyphenols and total catechins significantly decreased as the leaves matured and faded [99]. All five detected monomeric catechins were dramatically lowered in mature green leaves [99]. Higher expression levels of the EBGs in flavonoid synthesis in tender leaves support the idea of an elevated metabolic flux toward polyphenol accumulation [99]. Whereas, in the purple leaves of *Zijuan*, the abundance of EGCG is significantly lower, and the contents of EC, ECG, and EGC are much higher than those in green leaves [122]. According to the findings, the content alterations in the non-purple flavonoid components in diverse purple leaves do not exhibit a stable trend, potentially due to the highly complex regulation of genetic and environmental factors in this process.

## 4. Conclusions and Future Prospects

Leaf color variations give tea a unique appearance and nutritional quality. Under the current paradigm of homogenization in tea cultivars, varieties with leaf color variations typically have a higher economic value. Therefore, exploring the molecular mechanisms underlying leaf color formation will assist in the identification of mutation genes and also accelerate the utilization of leaf color variation varieties for tea breeding enhancement.

Using a systematic review of numerous omics investigations related to leaf color variation, we have concluded that the decrease in chlorophyll content and the accumulation of anthocyanin were the major common reasons for the color decoration of leaves in chlorina and purple-leaf tea varieties, respectively. In most chlorina tea varieties, the content and composition of carotenoids are altered, indicating that both photosynthetic activities and photooxidation protection capabilities are impaired in albinistic or etiolated leaves. The multi-omics results have also shown that decreased expression levels of critical genes in the biosynthesis of chlorophyll and carotenoids, activated chlorophyll degradation, and an impaired photosynthetic chain function are associated with the chlorina phenotype in tea plants. Decreased chlorophyll content not only impairs the structure and function of the chloroplasts but also leads to the degradation of the photosynthetic proteins that bind the plastid membrane. A decrease in the abundance of photosynthetic chain proteins also limits the content of bound chlorophyll and negatively influences its accumulation, as free chlorophyll is phytotoxic and unstable. Moreover, we have summarized that this tea plant chlorosis phenotype tends to result in the up-regulation of free amino acids and polar lipids and the inhibited accumulation of polyphenols. We have also proposed the possible causes of the changes in these bioactive metabolites in chlorina tea leaves. Overall, these metabolic changes in the chlorotic tissues embody a balance between leaf development and environmental adaptation, which fortunately endows the tea with a better flavor quality.

For purple-leaf tea varieties, the composition of purple-colored anthocyanin is affected by the tested environment and variety. The MBW complex plays a conserved upstream regulatory role in anthocyanin accumulation. The enhanced expression of LBG genes, especially those involved in the final two steps of anthocyanin synthesis, and positive changes in the genes related to the stable storage of anthocyanins play crucial roles in the formation of purple tea leaves. The decreased expression of genes associated with anthocyanin degradation may promote the accumulation of anthocyanins in some purple-leaf tea varieties. Additionally, heterosis of the purple-leaf phenotype is observed in the offspring of *Ziyan* and *Zijuan*, indicating the underlying genetic variations. The purple phenotype influences the accumulation of other flavonoids, which are also discussed in this paper.

While the results on the formation mechanisms and impact of the leaf color variation in tea can be identified according to certain macro patterns, the details of these physiological and molecular patterns vary greatly across different studies. The results indirectly suggest that these studies are likely limited to demonstrating the secondary outputs of leaf color variation, and the root causes of changes in leaf color or characteristic metabolite content remain poorly understood. Therefore, more in-depth studies must be carried out to uncover the mechanisms of leaf color formation in tea. (1) The function of candidate genes in tea plants should be verified using a transient transformation technology, such as a virus-induced gene-silencing system [123,124]. (2) Comparative omics research should select parents of leaf color variation materials as controls, reducing the interference from environmental conditions or developmental stages. (3) Genetic mapping and comparative genomics have been effective in identifying mutated genes in tea plants. Therefore, more efforts should focus on dissecting the genetic basis of leaf color variation and cloning mutated genes. (4) The influence of ecological or developmental factors on the metabolic flow allocation and the foundational molecular mechanisms should be explored. Through the above efforts, we will truly comprehend the mechanism of tea leaf color formation, explore its characteristic qualities more efficiently and accurately, and enhance the breeding utilization efficiency of these specific germplasms.

## Figures and Tables

**Table 1:** A summary of multi-omics-generated molecular evidence related to leaf color variations in tea.

Tea Varieties	Omics Approaches	Potential Molecular Mechanisms	References
*Anjibaicha* (Alternative names: *Baiye No1*, *White leaf No.1*, and *Anji white 1*)	Succinyl-proteome	Photosynthetic chain: The succinylation levels of PsbS and light-harvesting complex LHCA4 are down-regulated; the succinylation level of LHCB4 is up-regulated.	[46]
	Proteome and acetyl-proteome	Photosynthetic chain: lower abundance of LHCB1, LHCB2, LHCB3, LHCB4, LHCB5, LHCB7, LHCA1, LHCA2, LHCA3, LHCA4, PsbC, PsbD, PsbO, PsbP, PsbQ, PsbR, PsbS, Psb27, PsaE, PsaG, PsaL, PsaN, PetA, PetC, PetE, PetF, PetH, ATPA, ATPB, ATPD, ATPE, ATPG; a lower acetylation level of LHCA1.	[8]
	Pangenome	Chlorophyll synthesis: The *GluRS*/*EARS* gene in *Anjibaicha* showed a loss of anti-codon recognition domains; SVs were revealed in *ChlD*. Chlorophyll degradation: 1 bp deletion is revealed in *NOL*/*NYC1*.	[24]
	Whole-transcriptome	Chlorophyll synthesis: down-regulated expression of *POR* (two alleles), *CLH1*, *PBGD*/*HMBS*/*HEMC*; up-regulated expression of *COX15*. Photosynthetic chain: down-regulated expression of *CAB7*, *CAB21*, *LHCA4*, *CAB40* (three alleles), *CAB13*, *LHCB5*, *PetA* (two alleles), *PsbA* (*TEA_001460*), *PsaB*, *PsbP1*, *ATPA*, *PsaH*, ATPI, *PetB*, *Psb28*; up-regulated expression of *PsbA* (*TEA_001460*).	[35]
*Huangjinya*	Proteome	Chlorophyll synthesis: higher abundance of GluRS/EARS, MgCh/ChlH, and HMBS; lower abundance of POR. Photosynthetic chain: lower abundance of Photosystem Q(B) protein.	[36]
	Metabolome and proteome	Chlorophyll synthesis: higher abundance of GluRS/EARS and UROD/HEME; lower abundance of POR and CAO. Chlorophyll degradation: higher abundance of PAO/ACD1 Photosynthetic chain: lower abundance of LHCA1, LHCA3, LHCB1, LHCB2, LHCB3, LHCB4, LHCB5, LHCB6, PsaD, PsaF, PsaL, PsaN, PsbA, PsbD, PsbE, PsbO, PsbP, PsbQ; higher abundance of PsbS, FNR, ATPB. Carotenoid synthesis: lower abundance of ZEP; higher abundance of PSY.	[9]
	Pangenome	Chlorophyll synthesis: The *GluRS*/*EARS* gene in *Huangjinya* showed a loss of anti-codon recognition domains, which may inhibit chlorophyll synthesis; SVs were detected in *CAO*, *CHLP*, and *GluTR*. Carotenoid synthesis: mutated amino acids in CYP97A3^HJY^ and elevated expression of the *CYP97A3^HJY^* allele.	[24]
	Transcriptome	Carotenoid synthesis: down-regulated expression of *PSY*, *PDS*, *ZDS*, *LCYE*, *LCYB*, *CHY*, *ZEP*, and *VDE*.	[47]
*Baijiguan*	BSR-seq	Photosynthetic chain: co-down-regulated expression of *LHCA3*, three *LHCB1* alleles (*TEA001863*, *TEA001868*, *TEA030368*), two *LHCB3* alleles (*TEA017256*, *TEA021966*), and *LHCB4* in bulked groups and parents.	[29]
	Genome: Genotyping by sequencing and BSA-seq	Chlorophyll synthesis: a non-synonymous polymorphism (G1199A) in the magnesium chelatase I subunit (*CsChlI*).	[38]
*HY1*	Proteome	Photosynthetic chain: lower abundance of LHCA3, LHCA4, LHCB1, LHCB2, LHCB6, PsbC, PsBO, PsbS, PsaB, PsaC, PsaD, PsaF, PetA, PetH.	[17]
*HY2*	Proteome	Photosynthetic chain: lower abundance of LHCB2, PsbA, PsbD, PsbC, PsbB, PsbQ, PsaA, PsaB, PsaD, PsaH, PetB, PetA, beta F-type ATPase.	[17]
*HY*	Transcriptome	Chlorophyll degradation: the activation of *SGR* and *CLH*.	[43]
*Xiangfeihuangye*	Transcriptome, translatome, and metabolome	Chlorophyll synthesis: up-regulation of HY5 in EL inhibited the expression of *GluTR*/*HEMA* and *POR*.	[39]
*Huabai 1*	Transcriptome	Photosynthetic chain: down-regulated expression of light-harvesting complex II (*LHCII*) chlorophyll-a/b-binding protein. Chlorophyll degradation: up-regulated expression of *SGR*.	[12]
Albinistic branch of Huangshan	Transcriptome	Chlorophyll synthesis: down-regulated expression of four *CHLP* alleles (*TEA027589*, *TEA019124*, BGI_novel_G007262, *TEA016514*) and one POR allele (*TEA014780*); up-regulated expression of one *CHLP* allele (*TEA009538*), one *POR* allele (*TEA027994*), and one *CLH* allele (*TEA027808*). Photosynthetic chain: down-regulated expression of three *LHCB1* alleles (*TEA019232*, *TEA030366*, *TEA030368*), *PsbC*, five *PsbB* alleles (*TEA028468*, *TEA011113*, *TEA032780*, TEA018797, *BGI_novel_G013475*), *PsbP*, *Psb28*, *PsaA*, five *ATPD* alleles (*TEA030038*, *TEA004696*, *TEA002611*, *BGI_novel_G006800*, *BGI_novel_004911*), two ATPA alleles (*TEA019276*, *BGI_novel_G009498*), *ATPE*.	[48]
*Huangjinju*	Transcriptome	Chlorophyll synthesis: up-regulated expression of *POR*. Photosynthetic chain: down-regulated expression of *LHCA2*, *LHCA4*, *LHCB1*, *LHCB2*, *LHCB6*.	[13]
*Yanlingyinbiancha*	Transcriptome	Chlorophyll synthesis: down-regulated expression of *UROS*/*HEMD*, *PPOX*, *ChlH*/*GUN5*, *MgPEC*/*CRD1*, *DVR*/*PCB2*, and *CAO*. Chlorophyll degradation: down-regulated expression of *NOL*/*NYC1*, *HCAR*, *CLH1*, and *ACD2*. Photosynthetic chain: fifty-five DEGs involved in photosynthetic complexes were found to be down-regulated. Carotenoid synthesis: down-regulated expression of *Z-ISO*, *ZDS*, *ZEP*, *LUT2*, *NCED4*.	[18]
*Yanling Huayecha*	Transcriptome	Chlorophyll synthesis: down-regulated expression of *PPOX*. Photosynthetic chain: down-regulated expression of *LHCB6* and *FdC2*. Thylakoid membrane structure: down-regulated expression of *SCY1*.	[40]
*Menghai Huangye*	Transcriptome	Chlorophyll synthesis: four genes related to chlorophyll synthesis (*HEME2* and *POR*). Photosynthetic chain: ten genes related to photosynthesis (*LHCA* and *LHCB*) are down-regulated.	[41]
*Zhonghuang 3*	Transcriptome	Chlorophyll metabolism: down-regulated expression of *GluTR*/*HEMA3* and *CLH4*.	[49]
*Zhonghuang 3*	Transcriptome	Chlorophyll synthesis: down-regulated expression of *GluTR*/*HEMA*, *GSA-AM*/*HEML*, UROD/HEME, *HEMF*/*CPOX*, *DVR* (*CSS0009780*), and *CHLP*; up-regulated expression of *PBGS*/*HEMB*, *DVR* (*CSS0011936*), and *CLH*. Chlorophyll degradation: down-regulated expression of *NOL*/*NYC1* (*CSS0031926*) and *SGR* (*CSS0030812*); up-regulated expression of *NOL*/*NYC1* (*CSS0015127*), *SGR* (*CSS0050352*), and *SGRL* (*CSS0004139* and *CSS0036450*). Carotenoid synthesis: up-regulated expression of *Z-ISO*, CRTISO (*CSS0027469*, *CSS0033902*, and *CSS0044870*), *NCED1*, *NCED2*; down-regulated expression of *LCYB* and *NXS*.	[14]
*Zhonghuang 2*	Transcriptome	Transcripts encoding enzymes such as those functioning in early enzymatic steps, from the formation of glutamate 1-semialdehyde to protoporphyrin IX, showed lower levels. Critical enzymes for converting Mg-protoporphyrin IX into chlorophyll were also inhibited.	[42]
*Koganemidori*	Transcriptome	Chlorophyll synthesis: down-regulated expression of *POR*, *CAO*, and *ChlG*. Chlorophyll degradation: up-regulated expression of *CLH*. Transcriptional regulation: two homologs of *GLK* were significantly down-regulated.	[50]
*Huangjinshuixian*	Transcriptome and metabolome	Chlorophyll degradation: down-regulated expression of *SGR*. Carotenoid synthesis: the expression of *DXS* and *GGPPS* was significantly down-regulated. Transcriptional regulation: *PIFs* related to chlorophyll biosynthesis were significantly suppressed.	[16]
*Huangyu*	Transcriptome and metabolome	Chlorophyll synthesis: down-regulated expression of *UROD*/*HEME*, *MgCh*/*ChlH*, and *CAO*. Chlorophyll degradation: up-regulated expression of *CLH*. Photosynthetic chain: down-regulated expression of three *LHCII* genes (*CSS0013089*, *CSS0017825*, and *CSS0039893*)	[30]
*Huangkui*	Transcriptome	Transcriptional regulation: the transcriptional expression of *CsRVE1* increased during seasonal greening and was tightly correlated with increases in the expression of genes involved in light harvesting (*LHCB*) and chlorophyll biosynthesis (*MgCh*/*ChlH*, *GluTR*/*HEMA1*, and *CAO*).	[51]
*Fuhuang 1*	Transcriptome	Chlorophyll synthesis: down-regulated expression of *CAO*. Chlorophyll degradation: down-regulated expression of *NOL*/*NYC1* and *SGR*. Photosynthetic chain: down-regulated expression of *LHCA2*, *LHCA4*, *LHCB1*, *LHCB3*. Carotenoid synthesis: down-regulated expression of *LCYE*, *ZEP*, *NCED*; up-regulated expression of *PSY*, *PDS*, *VDE*.	[52]
*Fuhuang 2*	Transcriptome	Chlorophyll synthesis: down-regulated expression of *GluTR*/*HEMA*. Chlorophyll degradation: down-regulated expression of *CLH*. Photosynthetic chain: down-regulated expression of *PsbB*, *PetC*, *ATPF1B*, *LCHBs*, *LCHAs*. Carotenoid synthesis: down-regulated expression of *NCED*.	[53]

**Table 2:** A summary of anthocyanin composition in purple leaves of tea.

Tea Varieties	Sampling Location	Measurement Technique	Anthocyanin Composition	References
*Zijuan*	Tea garden of South China Agricultural University, Guangzhou, China	HPLC	Major anthocyanin compositions: cyanidin-3-*O*-galactoside and delphinidin-3-*O*-galactoside.	[77]
	Tea garden of the Institute of Tea Science, Yunnan Province Academy of Agricultural Sciences (Menghai, China)	Non-targeted metabolomics approach: UHPLC– Orbitrap–MS/MS	Cyanidin 3-diglucoside 5-glucoside, cyanidin 3-*O*-(6-*O*-*p*-coumaroyl) glucoside, cyanidin 3-sambubioside, cyanidin 3-(6″-acetylglucoside)-5-glucoside, delphinidin 3-(6-*p*-coumaroyl) galactoside, delphinidin-3-*O*-arabinoside, pelargonidin 3-sophoroside 5-glucoside, pelargonidin 3-coumarylglucoside-5-acetylglucoside, pelargonidin 3-rhamnoside 5-glucoside; compared with *Yunkang*, the contents of cyanidin 3-diglucoside 5-glucoside and pelargonidin 3-sophoroside 5-glucoside are most increased in *Zijuan*.	[86]
	Pu’er City Institute of Tea Science, Yunnan Province	UPLC–ESI–MS/MS metabolomic analysis	Specific metabolites: petunidin 3-O-glucoside, peonidin 3-O-glucoside chloride, peonidin 3-O-glucoside, peonidin O-hexoside, malvidin 3-O-glucoside (oenin), petunidin 3,5-O-diglucoside. Marker metabolites: cyanidin 3-O-galactoside, cyanidin 3-O-glucoside (Kuromanin), delphinidin 3-O-glucoside (Mirtillin), pelargonidin 3-O-glucoside	[87]
	Changsha, Hunan, China	UPLC–ESI–MS/MS metabolomic analysis	Major anthocyanin compositions: cyanidin-3-ogalactoside, delphinidin-3-O-galactoside, and petunidin-3-O-galactoside	[88]
	Dechang Fabrication Base of Shucheng County in Anhui Province, China	LC−TOF–MS	Cyanidin-3-O-galactoside, Cyanidin 3-O-(6-O-p-coumaroyl) galactoside, Delphinidin 3-O-(6-O-p-coumaroyl) galactoside, Delphinidin-3-O-galactoside.	[89]
*Zijuan Ziyan* and *Chuanzi* (ZZ)	Muchuan County, Sichuan Province, China	Targeted UPLC– ESI–MS/MS analysis	A total of 22 anthocyanins with a content ≥1 μg/g (DW) were detected in *Chuanzi*, *Ziyan*, and/or *Zijuan* and these included 6 cyanidins, 7 delphinidins, 5 pelargonidins, 2 peonidins, and 2 petunidins. In addition, 23 anthocyanins with a concentration of <1 μg/g were also detected.	[82]
*Ziyan*	Planted in plastic pots	HPLC	Delphinidin, cyanidin, and pelargonidin.	[90]
*Hongyecha*, *Zijuan*, *9803*, *Hongyafoshou*	Changsha, Hunan, China	UPLC–DAD–QTOF–MS	Cyanidin-(E)-p-coumaroylgalactoside, cyanidin-3-O-galactoside, delphinidin-3-O-galactoside, delphinidin-(Z)-p-coumaroylgalactoside, delphinidin-(E)-p-coumaroylgalactoside, pelargonidin-O-hexose, and pelargonidin-O-dihexose.	[91]
*Jinmingzao*	Tea plantation of Wuqu in Fuan City, Fujian Province, China	Widely targeted metabolomics: UPLC–ESI–MS/MS	Cyanidin 3-O-glucoside, cyanidin 3-O-galactoside, cyanidin 3-rutinoside, cyanidin chloride, delphinidin 3-O-glucoside, peonidin 3-O-glucoside chloride (most affected).	[83]
*Zikui*	South Campus of Guizhou University, Huaxi District, Guiyang City, Guizhou Province, China	ESI–QTRAP–MS/MS	Cyanidin 3-O-galactoside, cyanidin 3-O-glucosid, petunidin 3-O-glucoside.	[84]
*Longjing43*	Tea Research Institute, Chinese Academy of Agricultural Sciences, Hangzhou, China	LC–MS/MS	Delphinidin-hexose-coumaroyl showed the greatest increase.	[80]
*TRFK 306*	Tea Research Institute (TRI), Kericho County, Kenya	HPLC	Malvidin 3-glucoside, peonidin 3-glucoside, pelargonidin 3,5-O-diglucoside, cyanidin 3-O-glucoside, cyanidin 3-O-galactoside, cyanidin 3-O-rutinoside.	[81]
9 tea cultivars possessing purple leaves	Wuxi Institute of Tea Varieties in Wuxi City, Jiangsu Province, China	Widely targeted metabolomics: UPLC–ESI–MS/ MS	Thirty-three anthocyanins were identified, and delphinidin 3-O-galactoside and cyanidin 3-O-galactoside were found to be the most abundant in PTLs.	[85]
Unknown	Experimental tea farm (IHBT-269) of CSIR—Institute of Himalayan Bioresource Technology, HP, India	UHPLC	3-O-alpha-l-arabinopyranosylproantho cyanidin A5′ and 3,3′-Di-O-galloylprocyanidin B.	[92]

**Table 3:** A summary of multi-omics-generated molecular evidence related to purple leaves in tea.

Tea Varieties	Omics Approaches	Potential Molecular Mechanisms	References
*Zijuan*	Transcriptome	Transcriptional regulation: Activation of the R2R3-MYB transcription factor (TF) *anthocyanin1* (*CsAN1*) and the bHLH TF *CsGL3*; CsAN1 interacts with bHLH TFs (CsGL3 and CsEGL3) and recruits a WD-repeat protein CsTTG1 to form the MYB-bHLH-WDR (MBW) complex that regulates anthocyanin accumulation. Late biosynthetic genes (LBGs): activation of *CsF3′H*, *CsF3′5′H*, *CsDFR1*, *CsDFR2*, *CsANS1*/*LDOX1*, *CsANS2*/*LDOX2*, and *CsANS3*/*LDOX3*. Metabolic substrate competition: activation of *CsLAR1*, *CsLAR2*, and *CsLAR3*, which encode enzymes for catechin biosynthesis, was highly expressed in red foliage.	[77]
	Transcriptome, proteome	Phenylpropanoid metabolism: significantly increased expression of three *PALs* (*CSA016076*, *022024*, *022025*); significantly decreased expression of *4CL* (*CSA001434*). Early biosynthesis genes (EBGs): significantly increased expression of *CHS* (*CSA029775*); significantly decreased expression of *CHI* (*CSA008261*). LBGs: significantly increased expression of two *DFRs* (*CSA003949*, *XLOC_010242*), one *ANS*/*LDOX* (*CSA011508*), six *UGT75L12*/13 (*CSA005544*, *005545*, *010001*, *036671*, *036672*, *029026*), and two *UGT94P1* (*CSA007394*, *008750*); significantly decreased expression of *F3′5′H* (CSA031792), *ANS*/*LDOX* (*CSA035767*), two *UGT75L12s* (*CSA008693*, *028873*), and two *UGT94P1s* (*CSA005965*, *026000*). Metabolic substrate competition: significantly increased expression of two *LARs* (*CSA014943*, *XLOC_016774*).	[87]
	Transcriptome	Phenylpropanoid metabolism: activation of *C4H*. LBGs: activation of *ANS*/*LDOX*, *UGT*. Chlorophyll degradation: activation of *CLH1*.	[97]
	Full-length transcriptome	Alternative splicing (AS) events identified in transcriptional regulation (*MYB113-1*), phenylpropanoid metabolism (*C4H1*, *PAL2*), LBGs (*UDP75L122*), and metabolic substrate competition (*FLS1*).	[98]
	Proteome	EBGs: increased abundance of CHS and CHI. LBGs: increased abundance of DFR, ANS/LDOX, and UGT. Anthocyanin transportation: increased abundance of ABC transporter B8.	[20]
	Transcriptome	Transcriptional regulation: Most of the members belonging to the MYB, WRKY, AP2, GRF, bZIP, and MYC groups had a higher expression in *Zijuan*. LBGs: significantly increased expression of *F3′5′H* (*CSS0022212.1*), *ANS*/*LDOX* (*CSS0010687.1*), *3GT* (*anthocyanidin 3-O-glucosyltransferase*, *CSS0024320.1*), *3AT* (*cyanidin-3-O-glucoside 6″-O-acyltransferase*, *CSS0015285.1*). Metabolic substrate competition: significantly decreased expression of *LAR* (*CSS0009063*.1). Anthocyanin degradation: *polyphenol oxidase* (*PPO*, *CSS0002951.1*), showed negative correlation with the three anthocyanins, especially delphinidin and delargonidin.	[89]
*Chuanzi* (*ZZ*)	Transcriptome	Transcriptional regulation: significantly increased expression of the well-known MYB transcription factor *CsAN1/CsMYB75* (*CSS0030514*). LBGs: significantly increased expression of *CsANSs*/*LDOXs* (*CSS0010687*, *CSS0018498* and *CSS0046216*), *CsUGT94P1* (*CSS0011196*), and the *anthocyanin O-methyltransferase* gene (*CsAOMT*, *CSS0015915*). Anthocyanin transportation: significantly increased expression of *CsGSTF1* (*CSS0022086*) and three other GST candidate genes (*CSS0031248*, *CSS0026690,* and *CSS0018634*) tightly linked to CsGSTF1. Metabolic substrate competition: down-regulated expression of *LARs* (*CSS0028235* and *CSS0009063*) and *ANRs* (*CSS0005927*, and *CSS0033195*).	[82]
*Zijuan*, *Jinguanyin* and *Jinmingzao*	Pangenome	Read depth of the LTR insertion region in the promoter of *CsMYB114* among a set of representative purple-leaf cultivars (‘*ZJ*’, ‘*JMZ*’, and ‘*JGY*’) and tea cultivars with green leaves (‘*FDDB*’, ‘*BHZ*’, and ‘*GH3H*’)	[24]
*Ziyan*	Transcriptome	Transcriptional regulation: UV-A induces the expression of the regulatory gene *TT8*; UV-AB induces the expression of the regulatory genes *EGL1* and *TT2*. LBGs: UV-A induces the expression of *F3H*, *F3′5′H*, *DFR*, and *ANS*/*LDOX*; UV-AB induces the expression of *F3′5′H*, *DFR*, *ANS*/*LDOX*, and *UGT*. Metabolic substrate competition: UV radiation repressed the expression levels of *LAR*, *ANR*, and *FLS*, resulting in reduced ANR activity and a metabolic flux shift towards anthocyanin biosynthesis.	[90]
*Wuyiqizhong18*	cDNA-AFLP	EBGs: increased expression of *CHS*. LBGs: increased expression of *AT* (*TDF #3341_2f*) and *UGT* (*TDF #2421_1d* and *TDF #2411_1f*).	[79]
	Proteome	EBGs: increased abundance of CHS and CHI. Metabolic substrate competition: increased abundance of FLS.	[99]
*Jinmingzao*	Transcriptome	Phenylpropanoid metabolism: activation of *PAL*, *C4H*, and *4CL*. LBGs: activation of *DFR*, *ANS*/*LDOX*, and *UGT* (*TEA004632* and *TEA004632*) genes.	[83]
*Longjing43*	Transcriptome	Transcriptional regulation: activation of *MYB75*. LBGs: activation of *ANS*/*LDOX* and *3-GT*. Anthocyanin transportation: activation of genes involved in anthocyanin transportation (*GST*, *glutathione S-transferase*).	[96]
	Transcriptome	Phenylpropanoid metabolism: activation of *PAL* and *C4H* by high temperature and/or light levels in summer. EBGs: activation of *CHI* and *CHS* by high temperature and/or light levels in summer. LBGs: activation of *ANR*, *ANS*/*LDOX*, and *DFR* by high temperature and/or light levels in summer. Metabolic substrate competition: activation of *FLS* and *LAR* by high temperature and/or light levels in summer.	[80]
*TRFK 306*	Transcriptome	Transcriptional regulation: transcripts encoding pathway regulators of the MYB–bHLH–WD40 (MBW) complex were repressed, possibly contributing to the suppression of late biosynthetic genes of the pathway during the dry season. Anthocyanin transportation: suppression of anthocyanin transport genes could be linked to reduced accumulation of anthocyanin in the vacuole during the dry season.	[81]
*Zikui*	Transcriptome	Transcriptional regulation: *CsMYB90* showed strong correlations with petunidin 3-O-glucoside, cyanidin 3-O-galactoside, and cyanidin 3-O-glucosid. LBGs: activation of two *F3′H* genes and two *ANS*/*LDOX* genes. Anthocyanin degradation: three negatively correlated *PPO* (*polyphenol oxidase*) genes with anthocyanin accumulation.	[84]
*Hongyecha*, *Zijuan*, *9803*, *Hongyafoshou*	Transcriptome	Phenylpropanoid metabolism: activation of *4CL*. LBGs: activation of *ANS*/*LDOX* and *UGT*.	[88]

## Data Availability

All the data are contained within the article.
